# Exploring spatial-frequency-sequential relationships for motor imagery classification with recurrent neural network

**DOI:** 10.1186/s12859-018-2365-1

**Published:** 2018-09-29

**Authors:** Tian-jian Luo, Chang-le Zhou, Fei Chao

**Affiliations:** 1 Department of Cognitive Science, School of Information Science and Engineering, Xiamen University, 422 Siming South Road, Siming District, Xiamen, 361005 China; 2 Department of Computer Science, Institute of Mathematics, Physics and Computer Science, Aberystwyth University, Aberystwyth, Wales, SY23 3DB UK

**Keywords:** EEG signals classification, Spatial-frequency-sequential relationships, Deep recurrent neural networks, Brain computer interface

## Abstract

**Background:**

Conventional methods of motor imagery brain computer interfaces (MI-BCIs) suffer from the limited number of samples and simplified features, so as to produce poor performances with spatial-frequency features and shallow classifiers.

**Methods:**

Alternatively, this paper applies a deep recurrent neural network (RNN) with a sliding window cropping strategy (SWCS) to signal classification of MI-BCIs. The spatial-frequency features are first extracted by the filter bank common spatial pattern (FB-CSP) algorithm, and such features are cropped by the SWCS into time slices. By extracting spatial-frequency-sequential relationships, the cropped time slices are then fed into RNN for classification. In order to overcome the memory distractions, the commonly used gated recurrent unit (GRU) and long-short term memory (LSTM) unit are applied to the RNN architecture, and experimental results are used to determine which unit is more suitable for processing EEG signals.

**Results:**

Experimental results on common BCI benchmark datasets show that the spatial-frequency-sequential relationships outperform all other competing spatial-frequency methods. In particular, the proposed GRU-RNN architecture achieves the lowest misclassification rates on all BCI benchmark datasets.

**Conclusion:**

By introducing spatial-frequency-sequential relationships with cropping time slice samples, the proposed method gives a novel way to construct and model high accuracy and robustness MI-BCIs based on limited trials of EEG signals.

## Background

Motor imagery brain computer interfaces (MI-BCIs) construct path-ways by electroencephalography (EEG) signals’ event-related desynchronizing/event-related synchronizing (ERD/ERS) phenomenon in central brain’s band power in two rhythms, *μ* (8 - 12 Hz) and *β* (18 - 25 Hz) [1, 2]. Due to characteristics of EEG signals, conventional methods of MI-BCIs can be roughly divided into three categories: (1) classification by spatial features [3–7], (2) classification by frequency-spatial features [8–12], and (3) classification by temporal-frequency features [13–17]. The state-of-the-art approach of MI-BCIs was spatial-frequency features extracted by filter bank common spatial pattern algorithm (FB-CSP) [8, 12]. Such FB-CSP algorithm was effective for constructing optimal spatial features that discriminate among different classes of ERD/ERS rhythms in MI-BCIs by a bank of band-pass filters [18, 19]. By distinguishing the relationships between EEG signals and underlying primary source, the spatial-frequency features were good at solving the volume conduction effect [20].

Although the spatial-frequency features are enough for classification of EEG signals in MI-BCIs, the number of samples and simplified features are still two major challenges for the classification. First, since the conventional classification of EEG signals was usually adapted by “shallow” classifiers (linear discriminant analysis (LDA), support vector machine (SVM), and neural network (NN)) [21–25], such classifiers are appropriate for small sample size. Hence, a complete entity of each motor imagery trial’s spatial-frequency features was fed into these classifiers for classification. Due to the difficulty of obtaining motor imagery trials, public or private datasets have limited amounts of EEG trials from MI-BCIs [26, 27]. Thus, “shallow” classifiers with less data will produce poor performances of classification.

Second, except for spatial-frequency features, EEG signals’ sequential relationship is another useful feature for motor imagery classification. By cropping the spatial-frequency features into several time slices, each time slice can be treated as time-series, which contains sequential relationships over time. If the sequential relationships can be modeled by classifiers, the novel spatial-frequency-sequential relationships will significantly improve the performances and robustness of motor imagery classification.

To solve the two major challenges, this paper introduces a deep recurrent neural network (RNN) architecture for the classification based on FB-CSP algorithm [28, 29]. Also, by modeling EEG signals by RNN, an optimal number of hidden layers are obtained for RNN. Then, a sliding window cropping strategy (SWCS) is used to crop the entity trial into several time slices to increase the number of samples by the optimal number. Since the deep neural networks have dramatically improved the state-of-the-art methods in signal processing and classification, researches on EEG signals have been developed by using deep learning techniques to extract essential feature representations. The sequential relationships of EEG signals are easy to be extracted by RNN architecture. Therefore, the two contributions of this study are as follow:

1. A deep RNN architecture is applied to the FB-CSP features to extract the spatial-frequency-sequential relationships for motor imagery classification. The abundant features will improve the performances of classification. Also, two different memory units, long short-term memory (LSTM) unit [30] and gated recurrent unit (GRU) [31], are included in the RNN architecture.
2. The FB-CSP features extracted from a complete entity motor imagery trial are cropped by the SWCS with an optimal number. The strategy will increase a large a e deep neural networks.

## Related works

### Conventional methods

Manual feature extraction methods and shallow classifiers are developed for conventional motor imagery classification. These features are usually extracted from the spatial-frequency features and sequential relationships of EEG signals. Table 1 illustrates the related work regarding feature extraction methods and the corresponding classifiers in the state of the art methods.

**Table 1:** Conventional classification methods for motor imagery classification

Methods	Features	Classifiers	Datasets
Qin et al. (2004) [17]	Time-frequency	Source analysis	BCI competition II
Schlogl et al. (2005) [15]	AAR	kNN/LDA/SVM	BCI competiti on III
Herman et al. (2008) [22]	PSD	LDA/RFD/SVM	Graz University dataset
Blankertz et al. (2007) [21]	CSP	LDA	Graz University dataset
Ang et al. (2012) [8]	FB-CSP	PW Naive Bayes	BCI competition IV
Park et al. (2013) [25]	MEMD+CSP	SVM	BCI competition IV
Yuksel and Olmez (2015) [24]	SFN	BPNN	BCI competition III
Wu et al. (2015) [32]	P-CSP	FLDA	Paper’s private data
Kumar et al. (2018) [33]	Enhanced CSP	GA and SVM	BCI competition III and IV

From Table 1, we found CSPs algorithm [8, 21] is the key algorithm for extracting spatial features in motor imagery classification. Other researchers improve the CSPs algorithm by a probabilistic model [32] or the genetic algorithm (GA) [33]. Except for spatial features, the frequency features of power spectrum density (PSD) and sequential relationships of adaptive auto regression (AAR) are also used in motor imagery classification [22, 34]. Besides, the “time-frequency” features combine frequency features and sequential relationships for classification [17]. For the classification, conventional classifiers focus on shallow machine learning models. In some cases, the pre-processing algorithm multivariate empirical mode decomposition (MEMD) has been used to improve signal-noise ratio and classification accuracy [25]. The related works used manual features and shallow classifiers for the following reasons: on the one hand, because public datasets have limited EEG samples, they are more suited for classification by LDA/SVM/Naive Bayes classifiers; on the other hand, the EEG signals are regarded as a complete entity, and the entity is classified by spatial, frequency features or sequential relationships. However, if signals belong to time-series data, sequential relationships over time will provide the discriminant features for motor imagery classification.

### Deep learning methods

Statistical, integrated, and deep learning are the common classification methods in machine learning [35, 36]. In particular, deep learning classification methods have been used gradually for EEG signal classification [37–39]. Table 2 illustrates the related works regarding the state of the art of deep learning classifiers.

**Table 2:** Related works of EEG signal classification by deep learning

Methods	EEG phenomena	Deep learning architectures
Cecotti and Graeser (2008) [40]	Steady state visual evoked potential	CNN
Cecotti and Graser (2011) [41]	Event related potential 300ms	CNN
Yang et al. (2015) [43]	Motor Imagery	CNN
Kumar S and Sharma A (2016) [44]	Motor imagery	CSP+CNN
Hajinoroozi et al. (2015) [45]	Driver’s cognitive states	DBN
Wulsin et al. (2011) [46]	Abnormal EEG monitor	DBN
Zheng et al. (2014) [32]	Emotion	DBN
Ren and Wu (2014) [42]	Motor imagery	Convolution DBN
Forney and Anderson (2011) [47]	Imagined mental tasks	RNN
Soleymani et al. (2016) [48]	Durative affection	RNN

From Table 2, we conclude that deep learning is widely used in EEG signal classification. Convolution Neural Network (CNN) models [40–44] and Deep Belief Network (DBN) models [32, 45, 46] are most often used in the analysis of EEG signals. Actually, the CNN and DBN models are used to extract the spatial features from EEG signals. These two deep learning models still treat the complete entity trials for classification, so the performance can’t be improved much. However, the deep RNN architecture can extract the sequential relationships from EEG signals [47, 48]. By using a sliding window cropping strategy, the complete entity trials will be cropped into several time slices for classification. Several multiples growth number of samples by cropping for classification will obtain a significant performance improvement of motor imagery classification. Therefore, the discriminant features for motor imagery classification are extracted by using a combination of the FB-CSPs algorithm and RNN architecture.

## Methods

**Fig. 1:**
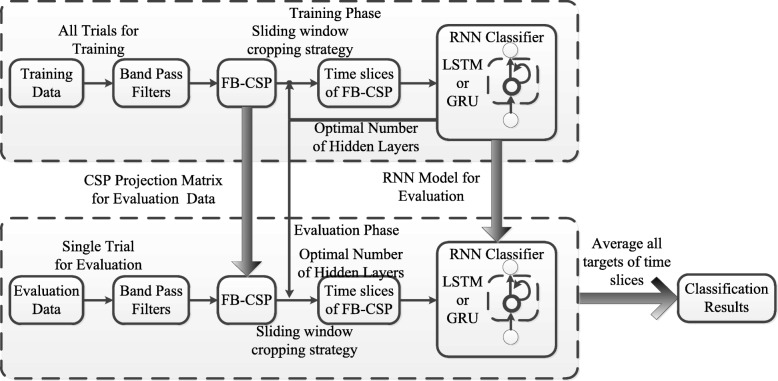
The procedure of our proposed methods. Our proposed method is comprised of four progressive stages of signal processing and machine learning on EEG signals: (1) a filter bank comprising multiple Butterworth band-pass filters to extract frequency features, (2) a CSP algorithm is used to extract spatial features, (3) a sliding window cropping strategy is applied to crop time slices to model the sequential relationships of spatial-frequency features, (4) classification of the spatial-frequency-sequential relationships on time slices by a deep RNN architecture. In the deep RNN architecture, two different memory units, LSTM unit and GRU, are included to compare classification performance and robustness

By considering references [47, 49], our proposed method regards EEG signals as time-series data, and the extracted spatial-frequency features’ sequential relationships are represented by RNN architecture. Due to the fact that conventional FB-CSPs algorithms with shallow classifiers do not contain the sequential relationships, and these algorithms regarded the entity of each trial as a single sample for classification. Therefore, two methods are developed to validate and represent spatial-frequency-sequential relationships for classification. First, we test a group of smoothing time windows on the FB-CSP features to validate whether the sequential relationships can improve the classification performance of EEG time-series. Then, a deep RNN architecture is applied to represent spatial-frequency-sequential relationships on FB-CSP features for classification. It is easy to cause over-fitting problems and drop the classification performance if the deep neural networks are presented for classification by the entity of a trial [50]. Therefore, before using the deep RNN architecture, a sliding window cropping strategy is applied to crop the entity of each trial into several time slices. Then, each time slice is fed into the deep RNN architecture for the motor imagery classification. The size of each time slice will be set as the same of the optimal number of hidden layers for RNN to obtain the optimal classification performance. The proposed method is illustrated in Fig. 1.

In Fig. 1, our proposed method is comprised of four progressive stages of signal processing and machine learning on EEG signals: (1) a filter bank comprising multiple Butterworth band-pass filters to extract frequency features, (2) a CSP algorithm is used to extract spatial features, (3) a sliding window cropping strategy is applied to crop time slices to model the sequential relationships of spatial-frequency features, (4) classification of the spatial-frequency-sequential relationships on time slices by a deep RNN architecture. In the deep RNN architecture, two different memory units, GRU and LSTM unit, are included to compare classification performance and robustness. The CSP projection matrix for each filter band, the discriminative spatial-frequency features, and the deep RNN architectures are computed and trained from training data labeled with the respective motor imagery action. These parameters computed from the training phase then used to validate each single-trial motor imagery action. By using the same cropping strategy in the validation phase, the classification of single-trial motor imagery action will predict several targets. The final evaluated action will be obtained by averaging all predicted targets.

### Spatial-frequency features

The widely used spatial-frequency features extraction algorithm for classification of motor imagery EEG signals was Filter Bank Common Spatial Patterns (FB-CSP) algorithm [8, 9]. There are two steps in the FB-CSP method: (1)a group of band-pass filters are presented to the raw EEG data to obtain the subject-specific frequency band. (2)The CSP algorithm is provided to every filter result to extract the optimal spatial features. Then, a classifier is used in all of the FB-CSP features for motor imagery classification.

To extract CSP features, let *X*_*c*_∈*R*^*N*∗*T*^ represent one band-pass filtering result, where *c* is the number of classes, *N* is the number of potentials of EEG, *T* is the number of samples in each trial. Each dataset contains *L* trials of EEG signals and each signal *X*_*c*_ is a zero average signal. The purpose of the CSP algorithm is to find an optimal spatial vector, $\overrightarrow w \in {R^{M \times \mbox{{N}}}}$, to project the original EEG signal to a new space to obtain good spatial resolution and discrimination between different classes of EEG signals. To calculate the optimal projection matrix, let the average covariance matrix of class “c” be $\overline C_{c}$, and average power of class “c” be ${\overline P_{c}}={\overrightarrow w^{T}}{\overline C_{c}}\overrightarrow w$. For an example of two classes on the minimized projected $\overrightarrow w$ axis, the maximization of the power ratio is written into the Rayleigh quotient form:

1 $$ \mathop {\arg \max }\limits_{\overrightarrow w} {\rm{ }}\frac{{{{\overline P }_{1}}}}{{{{\overline P }_{2}}}} = \mathop {\arg \max {\rm{ }}}\limits_{\overrightarrow w} \frac{{{{\overrightarrow w }^{T}}{{\overline C }_{1}}\overrightarrow w }}{{{{\overrightarrow w }^{T}}{{\overline C }_{2}}\overrightarrow w }}  $$

The Rayleigh quotient is then re-translated into a constrained optimization problem, which is then solved by applying the Lagrange multiplier method to the problem. The optimization results include both eigen-vectors and eigen-values. The optimal CSP spatial filter vector, ${\overrightarrow w^{*}} \in {R^{M \times N}}$, is constructed by taking *M*=2*m*,*M*≤*N* eigen-vectors corresponding to the “m” largest and “m” smallest eigen-values:

2 $$ {\overrightarrow w^{*}} = {\left[{\overrightarrow w_{{\lambda_{1}}}},\cdots,{\overrightarrow w_{{\lambda_{m}}}},\cdots,{\overrightarrow w_{{\lambda_{N - m + 1}}}},...,{\overrightarrow w_{{\lambda_{N}}}}\right]^{T}}  $$

where ${\overrightarrow w_{{\lambda _{i}}}}$ is the eigen-vector that corresponds to the eigen-value *λ*_*i*_. Each filter band of EEG signals, *X*_*c*_, is spatially filtered by:

3 $$ {Z_{c}} = {\overrightarrow w^{*}}{X_{c}}  $$

where *Z*_*c*_∈*R*^*M*×*T*^ is the spatial-frequency features. The EEG signals are composed of rapidly changing voltage values; therefore, band power (variance) is used as a feature for the classifier. For multi-class extension to the FB-CSP algorithm, the one-versus-rest (OVR) strategy is presented to solve the multi-class motor imagery BCI classification.

### Spatial-frequency-sequential relationships

Conventional algorithms for motor imagery EEG signals fed spatial-frequency features (FB-CSP) into classifiers to discriminate different motor imagery targets. In this paper, the FB-CSP features are fed into a deep RNN architecture to get spatial-frequency-sequential relationships to improve the classification performance of motor imagery. To validate and represent the spatial-frequency-sequential relationships, a group of smoothing time windows are put on the FB-CSP features to validate the effect of sequential relationships, and a RNN model with sliding window cropping strategy is applied to represent spatial-frequency-sequential relationships on EEG time-series. To improve the classification performance and overcome the over-fitting problem, the LSTM unit and GRU are used to construct LSTM-RNN architecture or GRU-RNN architecture for EEG signals classification.

#### Smoothing time windows on FB-CSP features

Since the FB-CSP features are extracted from EEG time-series, such features also contain sequential relationships. Before we represent the sequential relationships by the RNN architecture, a group of smoothing time windows are put on the FB-CSP features to smooth the sequential relationships. For the classification by FB-CSP features, we will adjust the smoothing time window size, and find the influence of classification performance by the smoothing time windows. If the influence for the performance is large, the sequential relationships on FB-CSP features will be validated to influence the classification performance. Therefore, the RNN architectures with LSTM and GRU memories will be applied to extract spatial-frequency-sequential relationships on FB-CSP features. According to the smoothing process, given smoothing window size, *ω*, the following smoothing operation is applied to the FB-CSP features, *Z*_*c*_:

4 $$ {\overline Z_{c}}(t) = {\frac{1}{\omega }\sum\limits_{n = 0}^{\omega} {{Z_{c}}(t - n)}}  $$

where ${\overline Z_{c}}(t)$ is the smoothed FB-CSP features. In the experiments, we adjust the parameter *ω* to obtain different smoothing levels of FB-CSP features, and get the classification performance by support vector machine (SVM). The classification performance will validate and instruct the sequential relationships for EEG signals classification.

#### RNN architectures with LSTM and GRU memories

**Fig. 2:**
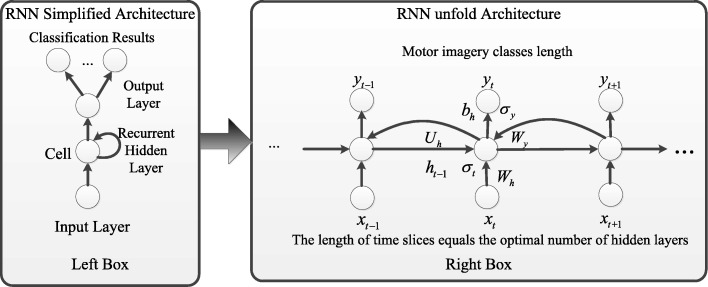
The standard deep RNN architecture. The simplified RNN architecture is shown in the box on the left. The box on the right shows the architecture unfolded in a form of time-series data [..., t - 1, t, t + 1,...]. Connections exist in the recurrent hidden layers; the input information of hidden layer “t” contains the output information of hidden layer “t+1”, and the sequential relationships over time are connected by horizontal lines

To represent the spatial-frequency-sequential relationships, we introduce the RNN architecture in this study [51, 52]. The RNN architecture, containing an input layer, recurrent hidden layers and an output layer, is widely used to represent time-series [53, 54]. In recurrent hidden layers, a number of simple computation units with weighted interconnections, including delayed feedback [28]. The feedback will give intrinsic states and learn tasks from memory, which is suitable for modeling EEG signals. With the activation functions, the deep RNN architecture is good at learning sequential patterns from EEG signals. Figure 2 illustrates the standard deep RNN architecture. In the figure, the simplified RNN architecture is shown in the box on the left. The box on the right shows the architecture unfolded in a form of time-series [⋯,*t*−1, *t*,*t*+1,⋯ ]. In the form of time-series hidden layers, the input of layer “*t*” contains the output of layer “ *t*+1”, so do the input of layer “ *t*−1”. The sequential relationships propagate from the end of the time-series to the start of the time-series by neurons, which are connected by horizontal lines in the figure.

Recurrent connections between hidden layers are followed by a feed-forward output layer. Hence, the deep RNN architecture is universal approximators of finite states. Therefore, a deep RNN architecture can approximate any finite states with enough recurrent hidden layers and trained weights. Let *Z*_*c*_∈*R*^*M*∗*T*^ represent FB-CSP features, where *M* is the features dimension, *T* is the number of samples in each trial. The RNN architecture can be defined as:

5 $$\begin{array}{*{20}l} {h_{t}} &= {\sigma_{t}}\left({W_{h}}{x_{t}} + {U_{h}}{h_{t - 1}} + {b_{h}}\right) \end{array} $$

6 $$\begin{array}{*{20}l}  {y_{t}} &= {\sigma_{y}}\left({W_{y}}{h_{t}} + {b_{y}}\right) \end{array} $$

where *x*_*t*_ is the vector of input layer, which is one of the time slices of the FB-CSP features *Z*_*c*_∈*R*^*M*∗*T*^. *h*_*t*_ is the vector of hidden layer. *y*_*t*_ is the vector of output layer. *W*, *U* and *b* are the recurrent connected weights. *σ* is the activation functions.

**Fig. 3:**
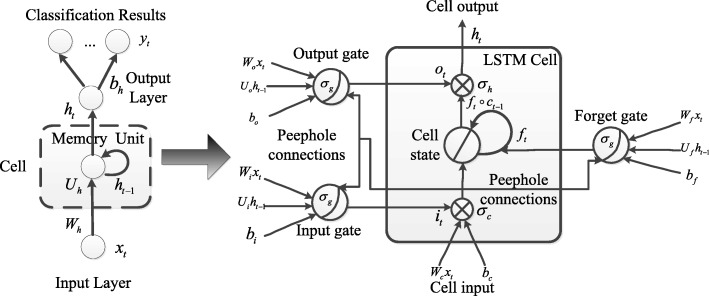
The LSTM unit architecture. In a LSTM unit, input, output and forget gates are used to retain memory contents; these gates also prevent the irrelevant inputs and outputs from entering the memory. Thus, the unit stores the long term memory features of the time-series data

Neural networks are processed by back-propagations (BP) algorithm in common. For the RNN architecture, the sequential relationships propagate all steps back through time, so the feedback of hidden layers will be processed by back-propagation through time (BPTT) algorithm [55]. The training procedure of a deep RNN architecture is performed using a stochastic gradient descent (SGD) algorithm. By using SGD algorithm, we can iteratively update the network’s weight values based on BPTT algorithm. However, the BPTT algorithm is too sensitive to recent distractions; thus, the error flow tends to vanish as long as the weights have absolute low variations, especially at the onset of the training phase. Long short-term memory (LSTM) unit [30] and Gated recurrent unit (GRU) [31] are proposed to overcome the vanishing gradient problem. The LSTM and GRU architecture is illustrated in Figs. 3 and 4. The introduction of these two architectures are as follow:

1. LSTM architecture: In a LSTM unit [56], input, output and forget gates are used to retain memory contents; these gates also prevent the irrelevant inputs and outputs from entering the memory. Thus, the unit stores the long term memory features of the time-series data. A peephole method [57] will be included in the LSTM architecture to transfer memories for all gates.

**Fig. 4:**
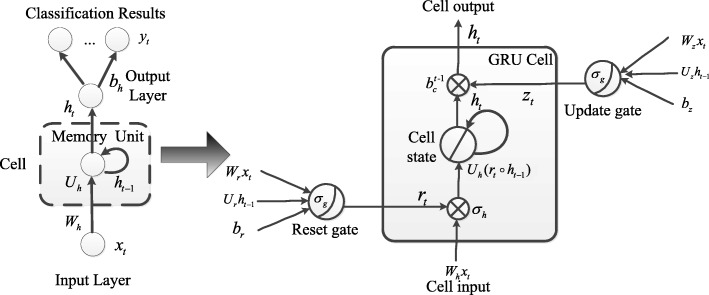
The GRU architecture. A GRU supports each recurrent unit to adaptively obtain dependencies of different time scales. The GRU has “update” and “reset” gates to prevent the error flow of information in the unit. Similarly to the LSTM unit, the gates prevent irrelevant inputs and outputs
2. GRU architecture: A GRU supports each recurrent unit to adaptively obtain dependencies of different time scales. The GRU has “update” and “reset” gates to prevent the error flow of information in the unit. Similarly to the LSTM unit, the gates prevent irrelevant inputs and outputs.

In such “memory units”, because these special units have internal states, multiplicative gates are employed to enforce constant error flow. These two different memory units are used in the deep RNN architecture to classify motor imagery tasks through spatial-frequency-sequential relationships. For each hidden layer of the RNN architecture, the original hidden layer will be replaced by LSTM unit or GRU to construct LSTM-RNN architecture or GRU-RNN architecture. Classification results are compared and analyzed to show which memory unit is more suitable for MI-BCI.

#### Sliding window cropping strategy

The conventional trial-wise EEG signals classification algorithms treat the entity duration of a trial as a single sample and the corresponding label as a single target. Then, a shallow classifier is used to train and validate motor imagery tasks. The conventional algorithms will lead to less samples and high dimensionality of features, which will cause the over-fitting problem and drop the accuracy of classification. In this study, a deep RNN architecture is used for the classification of EEG signals, if the entity duration of a trial is fed into deep RNN architecture, the number of hidden layers will be too large to get long-term patterns for the classification of EEG signals. To avoid the over-fitting problem of classification, a sliding window cropping strategy is applied to each trial to crop the entity duration of the trial into several time slices, and the label of the trial will be repeated to all time slices. This strategy will increase the number of training samples for the RNN architecture, which is widely used in the recognition tasks of image, audio and EEG signals by neural networks [58–60].

In our study, let *Z*_*c*_∈*R*^*M*∗*T*^ represents the inputs of RNN, the entity duration of a trial includes *T* time steps. Assumed *τ* is the cropping size of the sliding window cropping strategy, the time slices of the trial by cropping can be defined as

7 $$ {Z_{SWC}} \in \left\{ {Z_{t,\cdots,t + T}}|t \in 1,\cdots,T - {\tau}\right\}  $$

**Fig. 5:**
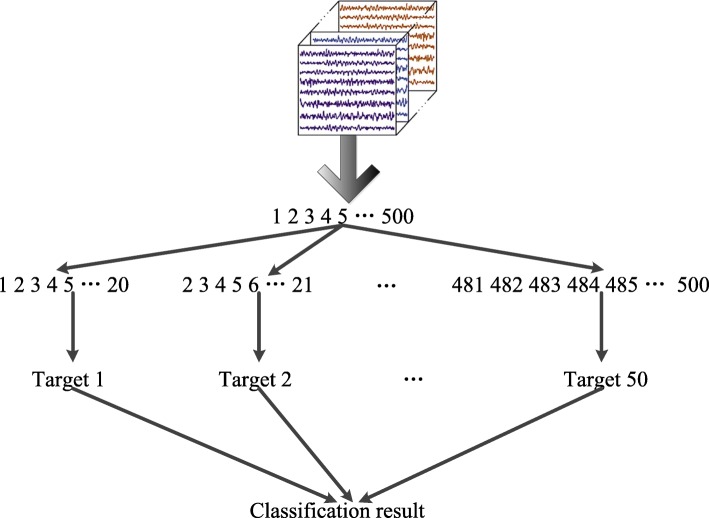
The sliding window procedure for cropping a trial into time slices. In common, the trial duration used for motor imagery is two seconds, and we obtain 500 samples for a 250 Hz sample rate. If the optimal number of the hidden layers is 20, the original trial will be crop to 480 time slices

The number of training samples will be increased *T*−*τ* times, and all time slices will get the label *y*_*c*_ as the same label from the original trial. Since the deep RNN architecture has the ability to extract signals’ sequential relationships for classification, we treat the number of hidden layers as the size of time slices. Therefore, we need to confirm the optimal number of hidden layers of the deep RNN architecture for motor imagery EEG signals classification; then, the optimal cropping size will be obtained from the EEG modeling experiment. If the optimal number of hidden layers is confirmed, the cropping size is confirmed. In common, the trial duration used for motor imagery is two seconds, and we obtain 500 samples for a 250 Hz sample rate. If the optimal number of the hidden layers is 20, the original trial will be crop to 480 time slices. The sliding window procedure for cropping a trial into time slices is shown in Fig. 5.

## Experiments and results

### Experimental datasets setup

**Fig. 6:**
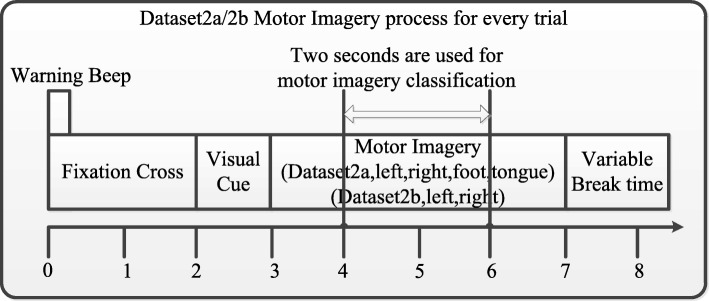
The procedure of single-trial motor imagery in BCI Competition IV. The BCI Competition IV experiments are composed of the following six steps: (1) Each trial started with a warning tone. (2) Simultaneously, a fixation cross was shown on the computer screen for two seconds. (3) After two seconds, a cue, in the form of an arrow, was randomly shown in lieu of the fixation cross, and the subjects started the corresponding motor imagery task of the cue. (4) After another 1.25 seconds, the cue reverted to the fixation cross. (5) The motor imagery task continued until the sixth second, at which time the fixation cross disappeared. (6) Finally, there was a short 1.5-seconds break

The performances of the algorithms were evaluated on the BCI Competition IV [27] “Dataset 2a” and “Dataset 2b”^1^. The two datasets are compared in Table 3. Figure 6 illustrates how the single-trial EEG data were extracted on “Dataset 2a” and “Dataset 2b”. The two datasets share the same procedure. In the motor imagery classification experiments, each subject sat in a soft chair comfortably facing a computer screen. The BCI Competition IV experiments are composed of the following six steps: (1) Each trial started with a warning tone. (2) Simultaneously, a fixation cross was shown on the computer screen for two seconds. (3) After two seconds, a cue, in the form of an arrow, was randomly shown in lieu of the fixation cross, and the subjects started the corresponding motor imagery task of the cue. (4) After another 1.25 s, the cue reverted to the fixation cross. (5) The motor imagery task continued until the sixth second, at which time the fixation cross disappeared. (6) Finally, there was a short 1.5 s break. The signals were sampled at 250 Hz and recorded. The pre-processing operations on the signals for notch filtered and band-pass filtered were 50Hz and 0.1-100Hz, respectively.

**Table 3:** Comparison of “Dataset 2a” and “Dataset 2b”

Dataset	Classes	Electrodes	Subjects	Sessions	Trials per session
2a	4(left, right, foot, tongue)	22EEG+3EOG	9	2	288
2b	2(left, right)	3EEG+3EOG	9	5	160

The BCI Competition IV “Dataset 2a” is composed of the following four classes of motor imagery EEG measurements from nine subjects: (1) left hand, (2) right hand, (3) feet, and (4) tongue. Two sessions, one for training and another for evaluation, were recorded from each subject. “Dataset 2b” is composed of two classes of motor imagery EEG measurements from nine subjects: (1) left hand and (2) right hand. Five sessions, the first three for training and the last two for evaluation, were recorded from each subject. According to the extraction procedure, the time range [4, 6s] was chosen for motor imagery classification because of a strong ERD/ERS phenomenon within that range [12, 44].

The spatial-frequency features are extracted by the FB-CSPs algorithm. In the division of the whole band (8-30Hz, covered *μ* and *β* rhythms) to obtain universality for all subjects, the optimal band width range is 4Hz overlaps the next by 2Hz [5, 25]. The optimal division of band-pass filters is shown in Table 4. After the optimal frequency bands filter the raw EEG signals, the CSP algorithm is applied to the filtered EEG signals to obtain spatial-frequency features. In (2) in the CSP algorithm, parameter *m* for processing “Dataset 2a” and “Dataset 2b” is set to 2 and 1, respectively.

**Table 4:** Optimal division of band-pass filters

Sub-bands	*f* *b*_1_	*f* *b*_2_	*f* *b*_3_	*f* *b*_4_	*f* *b*_5_	*f* *b*_6_	*f* *b*_7_	*f* *b*_8_	*f* *b*_9_	*f* *b*_10_
Frequency(Hz)	[8,12]	[10,14]	[12,16]	[14,18]	[16,20]	[18,22]	[20,24]	[22,26]	[24,28]	[26,30]

After extraction of spatial-frequency features, two separate experiments to confirm the parameters and validate the performances of spatial-frequency-sequential relationships and the classification of motor imagery are as follows:

1. EEG modeling experiments: First, a size range of [0, 4] smoothing time windows are put on the FB-CSP features to obtain the performance of classification. After validate the affections of performances by sequential relationships, two different sub-experiments on “Dataset 2a Subject 3” are presented to confirm whether a deep RNN architecture can model EEG signals well by cross-entropies and accuracies. Another sub-experiment is presented to find the optimal number of hidden layers in the deep RNN architecture.
2. Classification experiments: For motor imagery classification, the spatial-frequency FB-CSP features are fed into the deep RNN architecture to obtain spatial-frequency-sequential relationships. The spatial-frequency features are cropped by a sliding window sized by the optimal number of hidden layers. In the classification by LSTM-RNN architecture and GRU-RNN architecture, the accuracies, errors and efficiency of classification will be compared between spatial-frequency features and spatial-frequency-sequential relationships.

### EEG modeling experiments and results

**Fig. 7:**
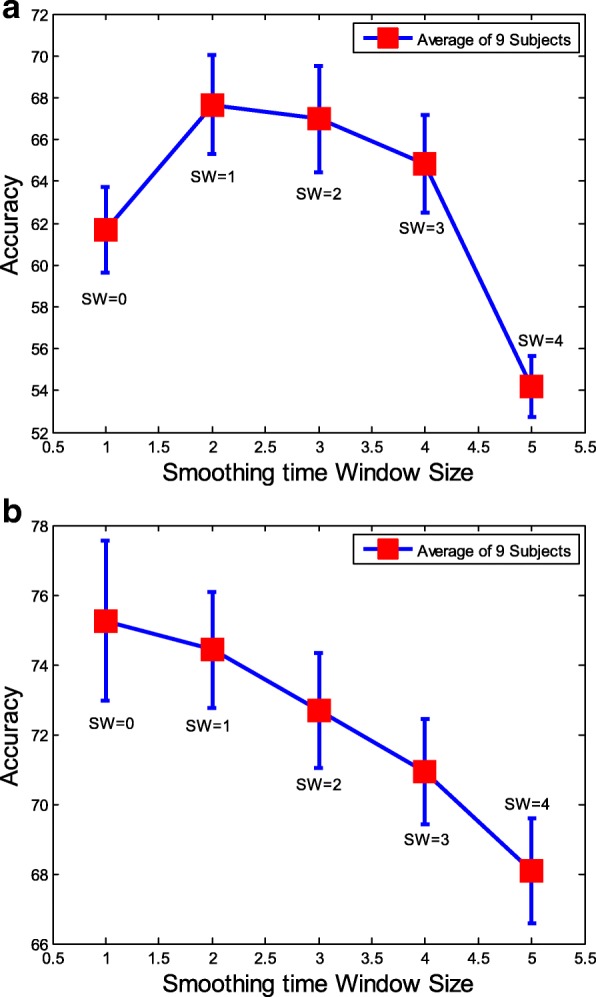
The classification results by using different size of smoothed FB-CSP features and SVM for both “Dataset2a” and “Dataset2b”. To obtain the performance of classification influenced by the sequential relationships, a group of smoothing windows with the size range [0,4] is presented to FB-CSP features. In our experiments, via smoothed FB-CSP features, the SVM classifier is used for motor imagery classification. Among the results, “SW=0” expresses the FB-CSP features without smoothing. The size number of smoothing time window fully influences the performance of EEG signals classification. **a** The classification results of “Dataset2a” and **b** The classification results of “Dataset2b”

To obtain the performance of classification influenced by the sequential relationships, a group of smoothing windows with the size range [0,4] is presented to FB-CSP features. In our experiments, via smoothed FB-CSP features, the SVM classifier with RBF kernel is used for motor imagery classification. Figure 7 illustrates the smoothing time window experimental results for “Dataset 2a” and “Dataset 2b”. Among the results, “SW=0” expresses the FB-CSP features without smoothing. From the results, we find that the performance of EEG signals classification was fully influenced by the smoothing time windows. Thus, the RNN architecture is introduced in this study to extract spatial-frequency-sequential relationships from FB-CSP features for classification. However, we must validate the presentation of spatial-frequency-sequential relationships by a RNN architecture at first.

**Fig. 8:**
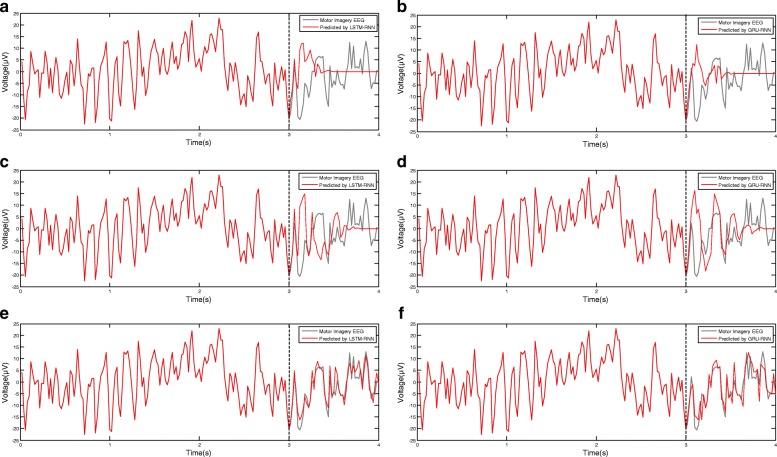
The prediction results of LSTM-RNN and GRU-RNN by 20, 30, and 90 hidden layers on channel “ *C*3”. The deep RNN architecture will predict the same level of signals as the number of hidden layers increased. A highest number of hidden layers will get rich sequential relationships which have a similar spectrum to the EEG signals. **a** 20 hidden layers of LSTM unit, **b** 20 hidden layers of GRU, **c** 30 hidden layers of LSTM unit, **d** 30 hidden layers of GRU, **e** 90 hidden layers of LSTM unit and **f** 90 hidden layers of GRU

There are three steps to validate the presentation of spatial-frequency-sequential relationships by RNN architecture. First, to validate whether the deep RNN architecture can model EEG signals or not, we train a deep RNN architecture by 200 iterations of SGD algorithm over 22 channels of the first three seconds of EEG signals from “Dataset 2a Subject 3”. To test the modeling ability, the previous outputs are fed back into model’s inputs to predict the current EEG signals. The results on channel “ *C*3” by 20, 30, 90 hidden layers are drawn in Fig. 8. From the results, we find the deep RNN architecture will predict the same level of signals as the number of hidden layers increased. The predictions by 20 hidden layers matched the EEG signals after a few samples, and the predictions by 30 hidden layers matched almost half of the rest samples. The predictions by 90 hidden layers matched the entity of rest samples for both LSTM-RNN architecture and GRU-RNN architecture. A highest number of hidden layers will get rich sequential relationships which have a similar spectrum to the EEG signals.

**Fig. 9:**
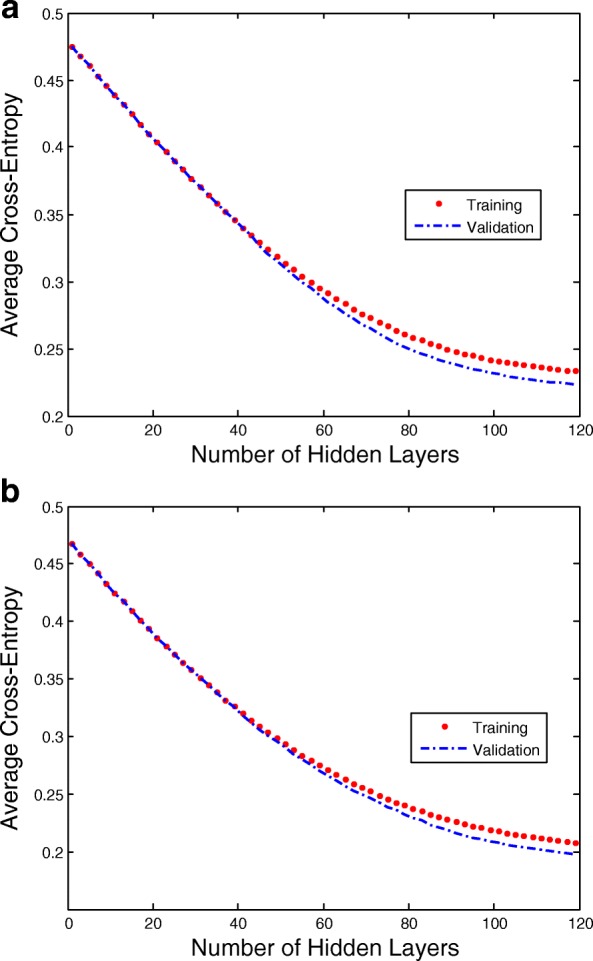
The curves of LSTM-RNN and GRU-RNN’s training and validation cross-entropies as the number of hidden layers increased. The training and validation cross-entropies have separations over 20 hidden layers. The cross-entropies will not reduce if the signals are over-fitting by RNN. In fact, the cross-entropies will not increase, so the deep RNN architecture continues to learn components of the signals that are common to all of the EEG sequences. **a** The curves of LSTM-RNN and **b** The curves of GRU-RNN

Second, we evaluate the classification performances of the deep RNN architecture by 200 iterations of SGD algorithm over the training data of “Dataset 2a Subject 3”. The loss function for EEG signals by RNN architecture is the logarithmic cross-entropy, which is defined as [61]:

8 $$ E = - \frac{1}{N}\sum\limits_{t = 1}^{T} {{c_{t}}\log b_{c}^{t} + (1 - {c_{t}})\log \left(1 - b_{c}^{t}\right)}  $$

where *c*_*t*_ is the ground truth result, and $b_{c}^{t}$ is the prediction result by deep classifier. The number of iterations for optimizing the loss function is an experience value of controlling training epochs by limiting the number of hidden layers. Figure 9 gives the training and validation cross-entropies as the number of hidden layers increased. From the results, we find the training and validation cross-entropies have separations over 20 hidden layers. The cross-entropies will not reduce if the signals are over-fitted by the RNN architecture. In fact, the cross-entropies will not increase, so the deep RNN architecture continues to learn components of the signals that are common to all of the EEG sequences. Compared with LSTM-RNN architecture and GRU-RNN architecture, the LSTM-RNN architecture needs more hidden layers to achieve a same level of cross-entropy during the classification of EEG signals.

**Fig. 10:**
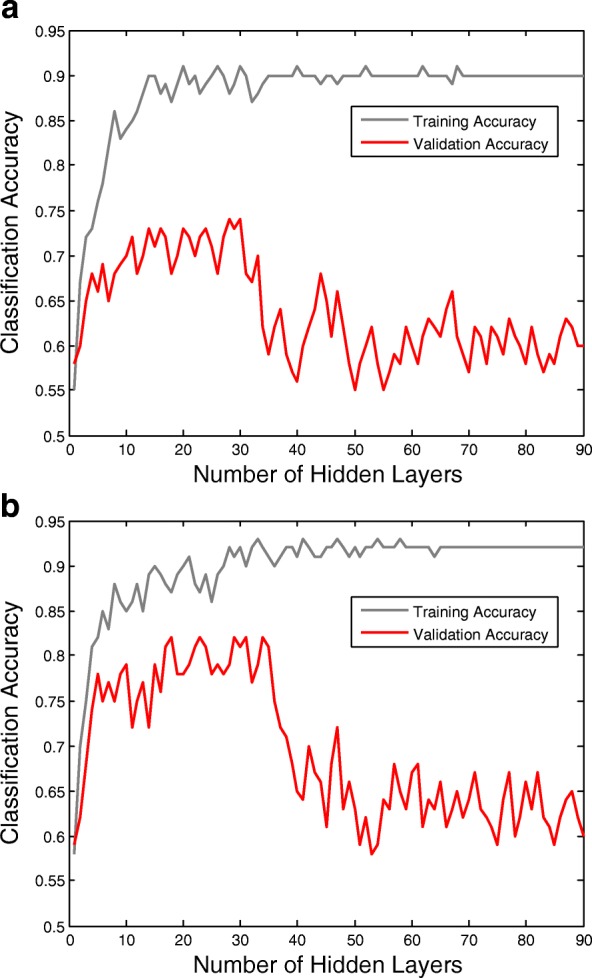
The curves of LSTM-RNN and GRU-RNN’s training and validation accuracies as the number of hidden layers increased. The validation accuracies appear peaks with a 20–15 hidden layers. The results of accuracies will be paradoxical with the results of cross-entropies, and the classification of EEG signals will be quickly over-fitting. The reason is that the deep RNN architecture continues to learn common components of the EEG sequences, while simultaneously learning signal noise and non-discriminative components. Therefore, there is a trade-off between classification and long-term patterns of modeling errors. **a** The curves of LSTM-RNN and **b** The curves of GRU-RNN

Third, since a large number of hidden layers requires much computational complexity, and causes the over-fitting problem to achieve low validation accuracies, Fig. 10 gives the training and validation accuracies as the number of hidden layers increased. From the results, we find the validation accuracies appear peaks with a 20–15 hidden layers. Compared with LSTM-RNN architecture and GRU-RNN architecture, the LSTM-RNN architecture needs more hidden layers to achieve a same level of accuracy during the classification of EEG signals. When the deep RNN architecture is over-fitting, the accuracy of GRU-RNN has a sharp drop than LSTM-RNN.

### Classification experiments and results

Let ${\overline Z_{c}} \in {R^{M * T}}$ represents the spatial-frequency features, where *M* is the feature dimension, *T* is the number of samples in each trial. After EEG modeling experiments, the optimal number of hidden layers for LSTM-RNN and GRU-RNN of all subjects are confirmed. Then, the optimal number *τ* is used for cropping training set and validation set by sliding window cropping strategy. Hence, the samples of each trial in training set and validation set will be increased *T*−*τ* times to satisfy the deep RNN architecture. After training procedure, the validation procedure will produce *T*−*τ* classification targets in each trial. Finally, the unique target of the trial will be calculated by averaging all targets of time slices.

To confirm the parameters and weights of RNN architecture, the characteristics of non-linearity and non-stationarity in EEG signals will be considered, since the characteristics will limit the reliability of the conventional activation function in the deep learning architecture. Therefore, there are three different activation strategies, “tanh”, “sigmoid” and “ReLu”, for constructing activation functions [62]. The activation function, “tanh”, is applied to cell input activation function of both the LSTM unit and GRU. The activation function, “sigmoid”, is applied to the cell output activation function of the LSTM units. To prevent the vanishing error flow, the “ReLu” activation function is applied to the gates activation function of both the LSTM unit and GRU. The weights of RNN are initialized by a Gaussian distribution *N*∼(0,0.2). The BPTT algorithm is used to train RNN by minimizing cross-entropy (see (8)) loss function. Also, because the Adam strategy [63] is suitable for time-series in deep classifiers and its momentum improves the robustness of error flow, the strategy is applied to compute the learning rate during BPTT. Finally, a “Dropout” strategy is applied to prevent the over-fitting problem [64]. The key idea of the “Dropout” strategy is to randomly eliminate units (along with their connections) from the neural network during training. By experience, the dropout rate is set at 0.2, and the maximum number of iterations is set at 200.

**Fig. 11:**
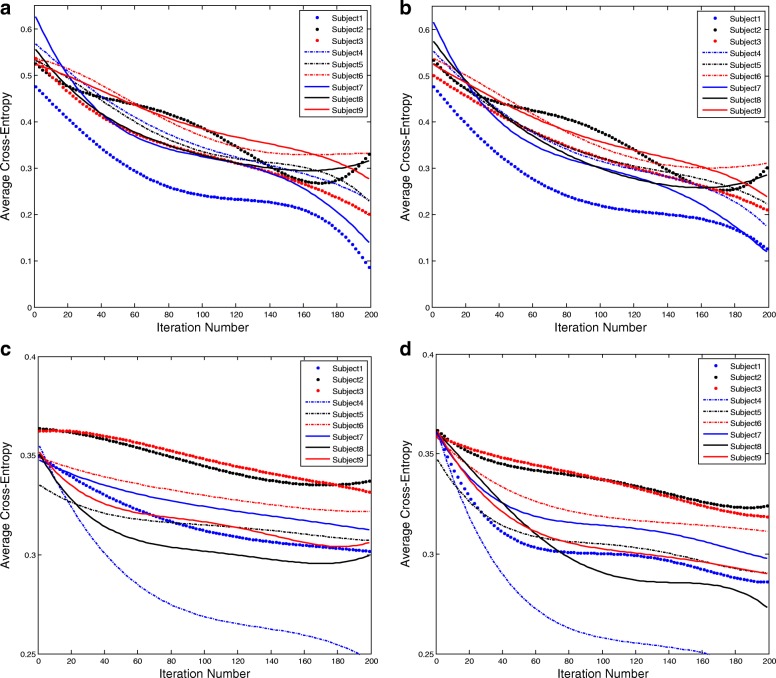
The learning curves for different memory units in different datasets. In the case of both datasets, GRU-RNN converge faster than LSTM-RNN. To reach lowest loss, GRU-RNN acquires less number of iterations than LSTM-RNN. For some specific subjects, GRU-RNN obtains lower average cross-entropy loss than LSTM-RNN within 200 iterations. Overall, the subjects’ EEG signals from “Dataset 2a” and “Dataset 2b” represent similar average cross-entropy between LSTM-RNN and GRU-RNN. **a** LSTM-RNN in “Dataset 2a”, **b** GRU-RNN in “Dataset 2a”, **c** LSTM-RNN in “Dataset 2b” and **d** GRU-RNN in “Dataset 2b”

For both “Dataset 2a” and “Dataset 2b”, we train two different RNN architectures, each of which includes LSTM unit and GRU. Figure 11 illustrates the learning curves for different memory units in different datasets. In the case of both datasets, GRU-RNN architecture converges faster than LSTM-RNN architecture. To reach lowest loss, GRU-RNN architecture acquire less number of iterations than LSTM-RNN architecture. For some specific subjects, GRU-RNN architecture obtains lower average cross-entropy loss than LSTM-RNN architecture within 200 iterations. Overall, the subjects’ EEG signals from “Dataset 2a” and “Dataset 2b” represent similar average cross-entropy between LSTM-RNN architecture and GRU-RNN architecture.

Table 5 gives the average training and validation time complexity per trial comparison between spatial-frequency-sequential relationships and spatial-frequency features. To compare time complexity, “Dataset 2a Subject 7” and “Dataset 2b Subject 8” are used to detect the average training and validation time complexity per trial. In Table 5, since the deep neural networks architectures (RNN and CNN) need more number of iterations for convergence in training phase, the average training time complexity of deep architectures is significantly higher than the conventional SVM model. However, in the validation phase, the deep architectures achieve a same level of time complexity than the conventional SVM model. Hence, the RNN architecture will cost appropriate time consumptions in the applications of MI-BCIs. Besides, compared with two different memory units of RNN architecture, GRU-RNN architecture outperform LSTM-RNN architecture in time complexity of EEG signals’ training and validation.

**Table 5:** Training and validation time complexity comparison between spatial-frequency- sequential features and spatial-frequency features with respect to “Dataset 2a Subject 7” and “Dataset 2b Subject 8”

Datasets and subjects		Spatial-frequency-sequential relationships		Spatial-frequency features	
		LSTM-RNN	GRU-RNN	CNN	SVM-linear
Training time	“Dataset 2a S7”	272.26	214.34	478.08	8.19
complexity (/s)	“Dataset 2b S8”	169.80	148.11	342.00	22.83
Validation time	“Dataset 2a S7”	1.89	1.49	3.32	2.01
complexity (/s)	“Dataset 2b S8”	3.40	2.96	6.84	4.17

All classification experimental results of all subjects in “Dataset 2a” and “Dataset 2b” are listed in Tables 6 and 7, respectively. Spatial-frequency-sequential relationships extracted from LSTM-RNN architecture and GRU-RNN architecture; spatial-frequency features extracted from CNN, SVM with linear, polynomial and RBF kernels are used to classify motor imagery for comparison. The results are presented by error rate forms, and a paired t-test statistical technique is used to detect whether the spatial-frequency-sequential relationships significantly outperform than spatial-frequency features in the classification of MI-BCIs. For each subject, we confirm the optimal number of hidden layers (ONHL) for LSTM-RNN architecture and GRU-RNN architecture.

**Table 6:** The misclassification rate and variance of motor imagery classification in “Dataset 2a”

Subjects	Spatial-frequency-sequential relationships					Spatial-frequency features		
	ONHL(*τ*)	LSTM-RNN(a)	ONHL(*τ*)	GRU-RNN(b)	CNN(c)	SVM-RBF(d)	SVM-polymonial(e)	SVM-linear(f)
S1	37	16.41 ±2.92	30	**15.18 ±2.86**	20.86 ±3.19	18.06 ±2.88	18.40 ±2.94	17.71 ±3.03
S2	35	**34.13 ±5.63**	29	34.68 ±5.56	48.18 ±6.87	43.40 ±6.69	46.53 ±6.55	46.18 ±6.36
S3	41	21.19 ±3.42	33	16.46 ±3.55	17.59 ±3.48	15.97 ±3.24	**15.28 ±3.35**	17.71 ±3.36
S4	40	**31.67 ±5.15**	31	32.33 ±5.60	44.12 ±7.37	39.24 ±5.64	37.15 ±5.48	42.71 ±7.19
S5	34	36.14 ±5.72	27	36.00 ±5.65	**32.53 ±5.28**	57.99 ±9.75	40.62 ±6.24	38.54 ±5.82
S6	36	33.11 ±5.15	26	**29.13 ±5.36**	49.30 ±8.68	46.87 ±8.98	47.22 ±8.52	47.57 ±8.49
S7	33	23.52 ±3.40	24	15.04 ±2.86	16.63 ±2.72	13.54 ±3.64	30.21 ±3.57	**12.16 ±2.40**
S8	36	23.60 ±3.63	28	28.05 ±3.75	**13.61 ±3.86**	29.51 ±4.35	28.13 ±4.63	31.94 ±5.72
S9	39	26.99 ±4.14	26	31.10 ±4.26	**15.71 ±3.85**	26.39 ±4.50	29.17 ±5.21	34.03 ±5.43
AVG	-	27.42 ±4.35	-	**26.44 ±4.38**	28.73 ±5.03	32.33 ±5.52	32.52 ±5.17	32.06 ±5.31
p-test	-	a vs. f	-	b vs. f	c vs. b	d vs. b	e vs. b	-
*p*-value	-	p=0.13	-	p <0.05^∗^	p=0.59	p=0.08	p <0.05^∗^	-

The smallest value for each subject is highlighted in bold. ^∗^:significant at 5% significance level for paired t-test

**Table 7:** The misclassification rate and variance of motor imagery classification in “Dataset 2b”

Subjects	Spatial-frequency-sequential relationships					Spatial-frequency features		
	ONHL(*τ*)	LSTM-RNN(a)	ONHL(*τ*)	GRU-RNN(b)	CNN(c)	SVM-RBF(d)	SVM-polynomial(e)	SVM-linear(f)
S1	39	22.61 ±4.63	31	**20.24 ±4.29**	30.66 ±5.96	35.94 ±6.46	32.5 ±5.27	33.44 ±5.65
S2	36	28.15 ±5.86	27	**27.24 ±5.69**	36.76 ±5.42	46.43 ±6.48	47.86 ±6.86	46.79 ±7.62
S3	44	27.87 ±4.53	35	**26.85 ±4.62**	38.61 ±5.27	45.94 ±7.52	44.69 ±7.40	45.62 ±7.65
S4	42	8.64 ±2.13	32	7.53 ±2.20	**1.87 ±1.03**	3.12 ±2.58	3.75 ±2.62	4.06 ±2.18
S5	36	14.67 ±3.46	28	**13.75 ±3.35**	14.58 ±3.28	19.37 ±4.25	16.25 ±3.86	16.25 ±3.97
S6	32	17.92 ±4.16	30	15.49 ±4.23	**12.64 ±3.60**	21.87 ±5.30	22.19 ±5.14	23.12 ±5.21
S7	33	14.53 ±3.59	39	13.35 ±3.46	**10.06 ±2.84**	22.81 ±4.72	22.50 ±4.41	22.81 ±4.23
S8	35	7.25 ±2.15	25	6.53 ±2.03	**3.16 ±1.86**	10.00 ±3.68	9.69 ±3.13	10.31 ±3.24
S9	38	24.64 ±4.62	28	**24.31 ±4.72**	30.66 ±5.13	17.19 ±3.42	16.56 ±3.35	17.19 ±3.41
AVG	-	18.48 ±3.90	-	**17.25 ±3.84**	19.89 ±3.83	24.74 ±4.93	24.00 ±4.67	24.40 ±4.80
p-test	-	a vs. f	-	b vs. f	c vs. b	d vs. b	e vs. b	-
*p*-value	-	p=0.08	-	p <0.05^∗^	p=0.28	p <0.05^∗^	p=0.06	-

The smallest value for each subject is highlighted in bold. ^∗^:significant at 5% significance level for paired t-test

**Fig. 12:**
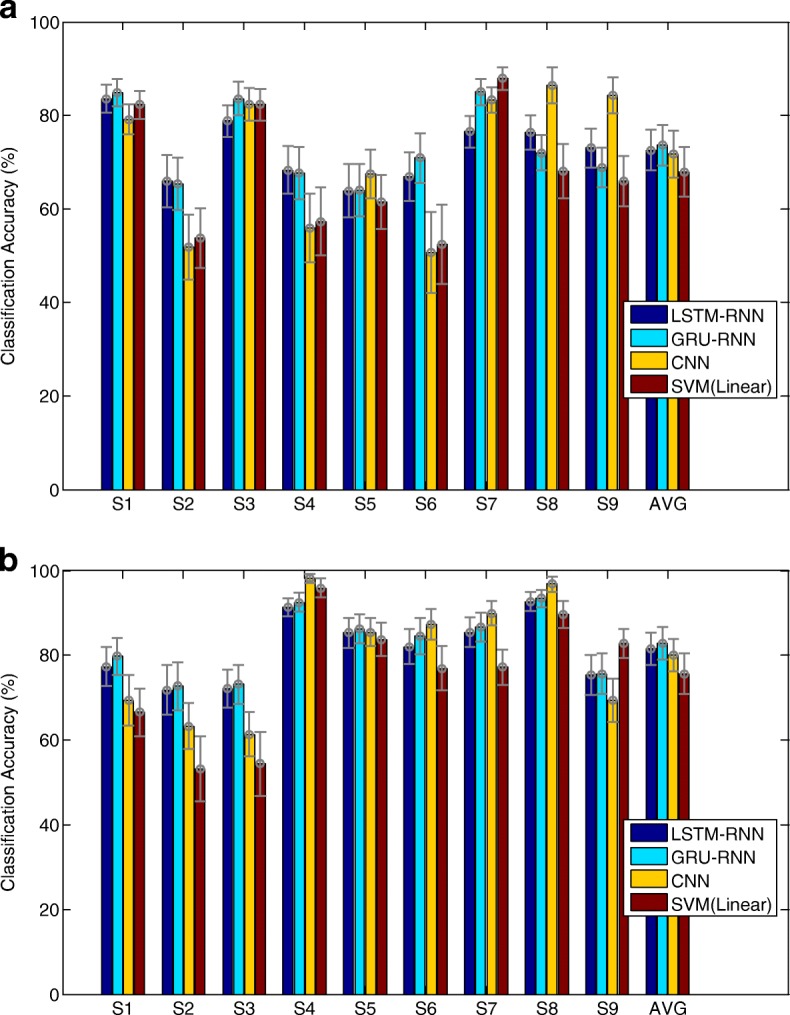
The classification accuracy performances for all subjects with all algorithms. CNN and SVM (Linear) outperforms RNN in some subjects with high-level (over 60%) accuracies (S3, S7, S8, S9 in “Dataset 2a” and S4, S6, S8, S9 in “Dataset 2b”). However, in low-level (below 60%) accuracies of subjects, RNN outperforms CNN and SVM-Linear (S2, S4, S6 in “Dataset 2a” and S2, S3 in for “Dataset 2b”). In the average-level accuracies, RNN outperforms CNN and SVM (Linear). **a** Classification accuracy performances for “Dataset 2a” and **b** Classification accuracy performances for “Dataset 2b”

From the results in Tables 6 and 7, for both datasets, spatial-frequency-sequential relationships outperform the spatial-frequency features in the classification of MI-BCIs. Among them, the average error rate of 27.42% and 26.44% is achieved by LSTM-RNN and GRU-RNN in the case of “Dataset 2a”, respectively. The results of paired t-test show GRU-RNN (b) achieves significantly lower error rate than SVM-Polynomial (e) **(p <0.05)** and SVM-Linear (f) **(p <0.05)**. In the case of “Dataset 2b”, an averaged error rate of 18.48% and 17.25% is achieved by LSTM-RNN and GRU-RNN, respectively. The results of paired t-test show GRU-RNN (b) achieves significantly lower error rate than SVM-RBF (d) **(p <0.05)** and SVM-Linear (f) **(p <0.05)**. To compare the classification performances of GRU-RNN and LSTM-RNN, Fig. 12 gives the classification accuracies for all subjects with all algorithms.

From the results in Fig. 12, we find CNN and SVM (Linear) outperformed RNN in some subjects with high-level (over 60%) accuracies (S3, S7, S8, S9 in “Dataset 2a” and S4, S6, S8, S9 in “Dataset 2b”). However, in low-level (below 60%) accuracies of subjects, RNN outperformed CNN and SVM (Linear) (S2, S4, S6 in “Dataset 2a” and S2, S3 in “Dataset 2b”). In the average-level accuracies, RNN architecture outperformed CNN and SVM (Linear). Besides, a comparison of the averaged accuracies for LSTM unit and GRU in both datasets shows that GRU-RNN architecture outperformed LSTM-RNN.

## Discussion

### Discussion for sequential relationships

Four different sub-experiments have been created to analyze the application of sequential relationships. From smoothing time window experimental results shown in Fig. 7, a small size of smoothing time window leaded to an improvement of classification accuracy for “Dataset 2a” and a decline of classification variance for “Dataset 2b”. However, a large size of smoothing time window leaded performance to decline for both “Dataset 2a” and “Dataset 2b”. The smoothing time window with different sliding sizes can change the sequential relationships; in addition, the experimental results demonstrated that the relationships significantly changed the classification performance; since the classification results can be changed if different sizes of smoothing time windows were applied to the sequential relationships. The finding gives us a novel enlightenment to smooth the extracted features to improve the classification performance and robustness [8, 12]. In addition, the sequential learning in the NLP also suggested to consider the sequential relationships as the key features for solving the natural language processing (NLP) problems [65, 66]. Therefore, due to the EEG signals contained the similar characteristics as the sentence structure in the NLP, the finding can assist us to use the sequential relationships to model the EEG signals.

In the experiments of representing EEG signals’ sequential relationships by RNN architectures, we found more hidden layers number of RNN architecture represented the EEG signals well, but more hidden layers number also caused memory vanishing problem(see Figs. 8, 9 and 10). To overcome the memory vanishing problem in the conventional RNN architecture, the LSTM-RNN architecture and GRU-RNN architecture have been introduced for the classification. We have validated the training and validation results in Figs. 9 and 10, the results of accuracies were paradoxical with the results of cross-entropies, and the classification of EEG signals will quickly over-fitting. The reason is that the deep RNN architecture continues to learn common components of the EEG sequences, while simultaneously learning signal noise and non-discriminative components [47]. Hence, here we must use propriate numbers of hidden layers to retain the classification performance. From the results in Fig. 10, the number of the hidden layers of the LSTM-RNN was about 30, and the number of hidden layers of GRU-RNN was about 35 [67, 68] (see Fig. 10). Therefore, the constraint of LSTM-RNN/GRU-RNN architecture leads us to crop the trial of EEG signals to time slices to feed into the classification architectures. Therefore, the SWCS is introduced on the time-series to crop the entity of a trial into several time slices. The time slices keep the same length of the number of hidden layers in order to well trained the LSTM-RNN architecture and GRU-RNN architecture.

### Discussion for EEG classification

In the EEG classification experiment, he results of RNN architectures outperformed the state-of-the-art methods (see Tables 6 and 7, and Fig. 12). There are two reasons for the results. The first reason is that EEG signals usually have easy distinguishing parts and difficult distinguishing parts. “Easy parts” for classification are well represented by the spatial-frequency features, since these features are statistical features. However, the “difficult parts” are nonlinear and non-stationary; therefore, the statistical features cannot well model these parts [69, 70]. Since the RNN architectures have enough neurons to fit the sequences’ nonlinear and non-stationary characteristics, the introduced spatial-frequency-sequential relationships retain the classification performance of “easy parts”, while improve the classification performance of “difficult parts”. The second reason is that the conventional spatial-frequency features regarded EEG signals as a complete entity, many factors can influence classification; in particular, the subject-specific diversity might be significant one. Instead of conventional features, spatial-frequency-sequential relationships consider the EEG signals as time-series to take spatial, frequency and temporal features of EEG data into consideration. This idea not only reduces the factors that influence classification, but also increases the corresponding robustness of the subjects by reducing the subject-specific diversity. Hence, the classification accuracies of MI-BCI by spatial-frequency-sequential relationships are significantly higher than those using the spatial-frequency features.

To solve the limitation of hidden layers number of RNN architecture, the SWCS is introduced on the time-series to crop the entity of a trial into several time slices. In this way, the number of samples for deep learning models is widely increased; therefore, enough samples are required to satisfy the generalization and performance of classification. In common, trials of EEG signals were obtained from complicated devices and the number of samples was too less to train the deep learning models. The application of SWCS solved the problem of sample number, and can also fit the RNN architecture. In addition, the LSTM and GRU are two different memory units for the RNN architecture. In order to test which memory unit is suited for processing EEG signals, the two memory units were applied to the RNN architectures. Our experimental results showed that GRU-RNN architecture was suited for the EEG signals better, and the spatial-frequency-sequential relationships with GRU memory units outperformed both shallow learning and deep learning models (see Table 5).

### Advantages of spatial-frequency-sequential relationships

Since the EEG signals are nonlinear and non-stationary signals, the spatial and frequency features are the robust statistical features, which can be well classified by SVM using FB-CSP features. We considered such features as the “easy parts” for classification; in contrast, we considered the temporal features as the “difficult parts” that are difficult to be classified by the conventional machine learning models. Hence, we introduced the spatial-frequency-sequential relationships by using RNN architecture on FB-CSP features. Experimental results showed that the approach involving spatial-frequency-sequential relationships can have the better classification performance of the “difficult parts”; also, the results outperformed the-state-of-the-art methods. Besides classifying the “difficult parts” of EEG signals, another reason for introducing the RNN architecture was that: the limitation number of hidden layers leaded us to crop the entity of a trial into several time slices. Although the different time slices shared the same labels, the slices increased the diversity of EEG signals during the classification. Therefore, the cropping strategy also improved the classification performance. To sum up, the advantages of introducing spatial-frequency-sequential relationships can improve classification performance and increase EEG samples diversity.

## Conclusion

In this paper, an FB-CSPs algorithm was used to extract spatial-frequency features, which were cropped by a sliding window cropping strategy into time slices. Then, the time slices were fed into deep RNN architectures, with two different memory units, to extract spatial-frequency-sequential relationships for MI-BCI classification. The extracted relationships included spatial, frequency and temporal characteristics. The experiments on MI-BCI demonstrated that the proposed method owned two advantages: (1) The spatial-frequency-sequential relationships extracted by FB-CSPs and RNN architectures can achieve significantly higher performance than spatial-frequency features. Meanwhile, the relationships had the same level of time complexity with the conventional algorithms. (2) A comparison of the accuracy and efficiency of motor imagery classification between GRUs and LSTM units revealed that GRUs can generate the better results.

Our future work will focus on collecting more EEG data to construct deeper RNNs architecture, exploring the error flow rules in BPTT, and constructing a deeper RNNs architecture that is adapted and generalized for EEG signals.

## Abbreviations

AAR: Adaptive auto regression
BPTT: Back-propagation through time
CNN: Convolution neural network
DBN: Deep belief network
EEG: Electroencephalography
ERD/ERS: Event-related desynchronizing/event-related synchronizing
FB-CSP: Filter bank common spatial pattern
GRU: Gated recurrent unit
LDA: Linear discriminant analysis
LSTM Long-short term memory; MEMD: Multivariate empirical mode decomposition
MI-BCIs: Motor imagery brain computer interfaces
NN: Neural network
OVR: One-versus-rest
PSD: Power spectrum density
RNN: Recurrent neural network
SVM: Support vector machine
SWC: Sliding window cropping
SWCS: Sliding window cropping strategy

## Footnotes

1
http://www.bbci.de/competition/iv/#datasets.
